# Observational study of haemostatic dysfunction and bleeding in neonates with hypoxic–ischaemic encephalopathy

**DOI:** 10.1136/bmjopen-2016-013787

**Published:** 2017-02-09

**Authors:** Mitali A Pakvasa, Anne M Winkler, Shannon E Hamrick, Cassandra D Josephson, Ravi M Patel

**Affiliations:** 1 Department of Pediatrics, Emory University School of Medicine, Atlanta, Georgia, USA; 2 Children's Healthcare of Atlanta, Atlanta, Georgia, USA; 3 Pathology and Laboratory Medicine, Emory University School of Medicine, Atlanta, Georgia, USA

**Keywords:** hemorrhage, hemostasis, hypothermia, transfusion

## Abstract

**Objective:**

Evaluate the relationship between initial haemostatic parameters and the frequency and severity of bleeding in neonates with hypoxic–ischaemic encephalopathy (HIE).

**Design:**

Retrospective observational cohort study.

**Setting:**

2 academically affiliated level III neonatal intensive care units in Atlanta, Georgia.

**Participants:**

98 neonates with moderate-to-severe HIE who underwent haemostatic testing within 12 hours of birth and were born from 1 January 2008 to 31 December 2013.

**Primary and secondary outcome measures:**

Initial haemostatic dysfunction was defined as one or more of the following: prothrombin time (PT) ≥18 s, platelet count <100×10^3^/μL or fibrinogen <150 mg/dL. Bleeding assessed using the Neonatal Bleeding Assessment Tool and graded according to the WHO bleeding scale. The robust Poisson regression was used to evaluate the independent association between components of initial haemostatic dysfunction and bleeding.

**Results:**

Among the 98 neonates evaluated, the prevalence of initial haemostatic dysfunction was 69% (95% CI 59% to 78%). 27 neonates (28%; 95% CI 19% to 38%) had abnormal bleeding events and 56 (57%) received at least 1 blood product transfusion. 3 neonates died from bleeding complications. The most common products transfused were fresh-frozen plasma (71%), followed by packed red blood cells (24%) and platelets (21%). In multivariable analysis, fibrinogen <150 mg/dL (adjusted relative risk 2.41, 95% CI 1.09 to 5.36) and platelet count <100×10^3^/μL (adjusted relative risk 2.59, 95% CI 1.30 to 5.16), but not initial PT, were associated with an increased risk of bleeding. The most severe bleeding occurred in neonates with a fibrinogen <150 mg/dL.

**Conclusions:**

Among neonates with moderate-to-severe HIE, haemostatic dysfunction is prevalent and associated with an increased risk of bleeding and high transfusion burden. Further studies are needed to determine the appropriate transfusion approaches in this population to prevent bleeding.

**Strengths and limitations of this study**

- This study estimated the incidence and quantified the location and severity of bleeding in neonates with hypoxic–ischaemic encephalopathy (HIE) using the NeoBAT, a standardised bleeding assessment tool for high-risk neonates.
- This study systematically evaluated the relationship between initial haemostatic dysfunction and the incidence, risk and severity of bleeding in infants with HIE.
- Study estimates of haemostatic dysfunction may be overestimated due to diagnostic bias, in which clinician-directed testing was more commonly performed in infants with greater illness severity.
- The association between initial haemostatic dysfunction and bleeding could have been biased by reverse causation, in which early bleeding resulted in haematological abnormalities.

## Introduction

Hypoxic–ischaemic encephalopathy (HIE) is a significant cause of neonatal morbidity and mortality and multiorgan dysfunction is common.^1^ ^2^ Disruption of haemostasis can occur from ischaemic injury, acute blood loss or disseminated intravascular coagulation.^3–6^ In neonates with HIE enrolled in clinical trials of therapeutic hypothermia, the incidence of bleeding ranged from 3% to 12%.^7^ ^8^ A more recent observational study reported a higher incidence of bleeding of 54% among neonates undergoing therapeutic hypothermia.^5^ In addition, the frequency of coagulopathy among neonates with HIE has also varied with reported prevalence ranging from 12% to 43%.^7–9^ Only one prior study has evaluated the association between coagulation parameters and abnormal bleeding events in this population.^5^ In this study, thresholds of platelet count <130×10^3^/µL, fibrinogen activity <1.5 g/L and international normalised ratio (INR) >2.0 discriminated between neonates with and without bleeding. However, this study did not specifically evaluate the prevalence of initial haemostatic function. Additionally, no studies have graded the severity of bleeding in neonates with HIE and there are limited data to guide transfusion therapy, despite the common administration of blood products in this population.

Understanding haemostatic dysfunction among neonates with HIE may help guide risk assessment and transfusion practices in this high-risk population. We sought to estimate the prevalence of initial haemostatic dysfunction in neonates with moderate-to-severe HIE and to characterise the incidence, severity and location of bleeding. In addition, we evaluated the relationship between specific components of initial haemostatic dysfunction and the frequency and severity of bleeding events.

## Methods

### Patient population

This retrospective cohort study was performed at two academically affiliated level III neonatal intensive care units in Atlanta, Georgia, USA (Grady Memorial Hospital, Emory University Hospital Midtown). All consecutively admitted neonates born between 1 January 2008 and 31 December 2013 were eligible for inclusion if they met the following three criteria: (1) birth weight ≥1800 g; (2) moderate-to-severe HIE; (3) testing of any haemostatic parameter within 12 hours of birth. Exclusion criteria included no initial haemostatic testing or testing after 12 hours of life. Appropriate oversight and approval was obtained from the Emory University Institutional Review Board and Grady Memorial Hospital Research Oversight Committee. The study was reported according to the STrengthening the Reporting of OBservational studies in Epidemiology (STROBE) statement.^10^

### Definitions

Haemostatic dysfunction was defined as the presence of coagulopathy, thrombocytopenia or hypofibrinogenaemia (defined below) occurring within 12 hours of birth. Reagents used by the hospital coagulation laboratory did not have neonatal-specific reference ranges. Therefore, we selected a prothrombin time (PT) of 18 s, a priori*,* to define coagulopathy, taking into consideration the values from published normative ranges,^11^ with some allowance for mild elevation. We chose to use PT as opposed to the INR to define coagulopathy, as INR was inconsistently reported or recorded at study sites. We also evaluated the sensitivity of using a cut-point by evaluating PT prolongation as a continuous variable. Thrombocytopenia was defined as a platelet count <100×10^3^/μL based on this value as the trigger for the upper threshold for platelet transfusion among neonatologists.^12^ ^13^ Hypofibrinogenaemia was defined using the cut-point of <150 mg/dL, based on this value being the lower limit of normative ranges in healthy term newborns at birth and recently published data among neonates with HIE receiving therapeutic hypothermia.^5^ Haemostatic parameters outside of the diagnostic range were assigned to the upper or lower value of the detection limit. Moderate-to-severe HIE was determined using criteria from Neonatal Research Network trials.^8^ We evaluated all blood products transfused during the first 96 hours after birth. The decision to transfuse was at the decision of the treating neonatal caregivers, as the hospitals in this study did not have transfusion protocols or guidelines for this population of infants. Outcomes were compared between those neonates who experienced an abnormal bleeding event versus those who did not. We ascertained bleeding events during the first 96 hours after birth based on a diagnosis of or notation of abnormal bleeding in physician, nursing or respiratory therapist documentation. We also adapted the Neonatal Bleeding Assessment Tool (NeoBAT), a validated bleeding assessment tool, to retrospectively assess the severity of abnormal bleeding events.^14^ We used the modified NeoBAT to ascribe all patients with bleeding events a modified WHO bleeding assessment scale score (scale of 1–4, with 4 being the most severe). Patients without bleeding were assigned a score of 0.

### Statistical analysis

SPSS 22.0 for Windows (IBM, Armonk, New York, USA) was used for all statistical analysis. Data acquisition and statistical analysis was performed from 22 October 2014 to 6 May 2016. As this study was a convenience sample, no sample size estimation was performed. Normally distributed continuous variables were described using means and SDs and compared using the Student's t-test. Medians with IQRs were used for non-normally distributed variables and compared using the Wilcoxon rank-sum tests. Categorical variables were compared using the χ^2^ or Fisher's exact tests. Binomial CIs were obtained using the Clopper and Pearson method. Denominators were specified to indicate missing data, when present; imputation was not performed. Relationships between PT, fibrinogen, platelet count and bleeding severity were characterised using a bubble plot. Linear trends between ordinal categories and response variables were evaluated using generalised linear models. The ability of initial coagulation parameters to discriminate bleeding and non-bleeding neonates was determined using standard measures of test performance with clinically determined and previously published cut-points.^5^ We also evaluated the performance of parameters in multivariable logistic regression models to discriminate bleeding versus non-bleeding neonates using the area under the curve.

The frequency of abnormal bleeding events was compared by the individual components of initial haemostatic dysfunction. The Poisson regression with robust SEs was used to evaluate the association between initial haemostatic dysfunction and abnormal bleeding events. Baseline haemostatic parameters were included in multivariable regression models, to determine the independent association of bleeding with individual haemostatic parameters. Only main effects were specified in the model. No interaction testing was performed in order to limit parameters to 1 per 10 outcome events to protect against overfitting. Because of the right-tailed distribution of PT and significant correlation between PT, platelet count and fibrinogen (Spearman's correlation coefficients of 0.44–0.54), we specified these values as categorical variables in the multivariable model. PT was categorised using approximate tertiles, with the first tertile consistent with our prespecified definition of coagulopathy. Activated partial thromboplastin time (APTT) and INR were not included, given the collinearity with PT.

## Results

Over a 72-month period, we reviewed medical records of 505 neonates who weighed 1800 g or more at birth and had either a 5 min APGAR ≤7 or admission diagnosis of HIE. Of these, 132 were diagnosed with moderate-to-severe HIE and 98 neonates underwent haemostatic testing within 12 hours of birth and were included in the study for further analysis. Of note, we found no difference in the frequency of severe HIE (36% vs 29%, p=0.50), bleeding (28% vs 24%; p=0.65) or receipt of therapeutic hypothermia (98% vs 94%, p=0.16) between neonates who underwent initial haemostatic testing within 12 hours of birth and were included in the study (n=98) and those neonates who did not meet this inclusion criterion (n=34).

### Patient characteristics

We compared neonates with and without abnormal bleeding events. There were no significant differences in the mean birth weight or gestational age between neonates with and without bleeding (table 1). There were no significant differences in sex, hospital of birth, severity of HIE or 1 and 5 min Apgar scores between groups. Neonates with bleeding, compared with non-bleeding neonates, were more likely to have received saline during delivery room resuscitation (41% vs 19%, p=0.02). There was also no significant difference in maternal trauma, uterine rupture, shoulder dystocia or maternal placental abruption between neonates with and without bleeding. Ninety-nine per cent of neonates in the study received therapeutic hypothermia.

**Table 1:** Maternal and neonatal characteristics

	No bleeding	Bleeding	
Maternal characteristics	n=71	n=27	p Value
Maternal age (years)	28±7	31±5	0.03
Race			0.96
White	20/69 (29%)	6/24 (25%)	
Black	34/69 (49%)	12/24 (50%)	
Hispanic	11/69 (16%)	4/24 (17%)	
Other	4/69 (6%)	2/24 (8%)	
Caesarean delivery	52/71 (73%)	22/27 (81%)	0.40
Severe trauma	1/71 (1%)	0/27 (0%)	>0.99
Placental abruption	14/71 (20%)	9/27 (33%)	0.16
Uterine rupture	6/71 (8%)	6/27 (22%)	0.09
Shoulder dystocia	2/70 (3%)	1/27 (4%)	>0.99
**Neonatal characteristics**	**No bleeding**	**Bleeding**	p Value
Birth weight (g)	3200±606	3390±690	0.19
Gestational age (weeks)	38.7±1.7	38.9±1.7	0.69
Female	29/71 (41%)	11/27 (41%)	0.99
Inborn	52/71 (73%)	18/27 (67%)	0.52
1 min Apgar, median (IQR)	1 (0–2)	1 (0–1)	0.23
5 min Apgar, median (IQR)	3 (1–4)	3 (0–4)	0.31
Delivery room care			
Intubation	64/70 (91%)	23/27 (85%)	0.46
Chest compressions	32/70 (46%)	16/27 (59%)	0.23
Epinephrine	23/70 (33%)	14/27 (52%)	0.08
Saline administration	13/70 (19%)	11/27 (41%)	0.02
RBC administration	0/70 (0%)	0/27 (0%)	>0.99
Cord blood gas pH	6.88±0.21	6.85±0.19	0.67
Admission blood gas pH	7.03±0.23	6.99±0.22	0.52
Severity of HIE			
Moderate	49/71 (69%)	14/27 (52%)	0.11
Severe	22/71 (31%)	13/27 (48%)	
Therapeutic hypothermia	70/71 (99%)	27/27 (100%)	>0.99
Death (in hospital)	8/71 (11%)	6/27 (22%)	0.20
Death from haemorrhage	0/71 (0%)	3/27 (11%)	0.02

Values listed are n/N (%) or mean±SD, unless indicated otherwise.

Missing values for continuous variables: maternal age, 6; gestational age, 1; 1 min Apgar, 2; 5 min Apgar, 2; cord blood gas pH, 39; admission blood gas pH, 14.

HIE, hypoxic–ischaemic encephalopathy; RBC, red blood cell.

### Coagulation abnormalities and transfusion burden

The prevalence of initial haemostatic dysfunction was 69% (95% CI 59% to 78%), largely accounted for by the high prevalence of a prolonged PT ≥18 s. Neonates with bleeding had significantly higher initial PT, APTT and INR values, and lower fibrinogen activity platelet counts than neonates without bleeding (table 2). The most common blood products transfused in the first 96 hours after birth were fresh-frozen plasma, followed by packed red blood cells (table 2). Although the receipt of any blood product transfusion was more common among neonates with bleeding, approximately half of neonates without bleeding received at least one blood product transfusion. Platelet transfusion occurred only among neonates with bleeding.

**Table 2:** Haemostatic and transfusion characteristics among bleeding and non-bleeding neonates

	No bleeding	Bleeding	
Haemostatic characteristics	n=71	n=27	p Value
PT (s) (n=95)	19 (17–26)	28 (18–58)	0.004
APTT (s) (n=74)	54 (45–63)	72 (51–135)	0.005
INR (n=82)	1.7 (1.4–2.4)	2.7 (1.6–4.4)	0.008
Fibrinogen (mg/dL) (n=83)	163 (135–199)	80 (70–141)	<0.001
Initial platelet count (10^3^/μL) (n=93)	190 (160–235)	156 (98–174)	0.001
PT ≥ 18 s	43/69 (62%)	23/26 (89%)	0.01
Fibrinogen <100 mg/dL	7/61 (12%)	13/22 (59%)	<0.001
Fibrinogen <150 mg/dL	22/61 (36%)	17/22 (77%)	0.001
Initial platelet count <100×10^3^/μL	3/67 (5%)	7/26 (27%)	0.004
**Transfusion characteristics**			
*Frequency of blood product transfusions*			
Packed red blood cell			
None	58/71 (82%)	17/27 (63%)	0.049
1	10/71 (14%)	5/27 (19%)	
2 or greater	3/71 (4%)	5/27 (19%)	
Platelet			
None	71/71 (100%)	20/27 (74%)	<0.001
1	0/71 (0%)	3/27 (11%)	
2 or greater	0/71 (0%)	4/27 (15%)	
Fresh-frozen plasma			
None	41/71 (58%)	9/27 (33%)	0.002
1	16/71 (23%)	3/27 (11%)	
2 or greater	14/71 (20%)	15/27 (56%)	
Cryoprecipitate			
None	70/71 (99%)	24/27 (90%)	0.08
1	1/71 (1%)	2/27 (7%)	
2 or greater	0/71 (0%)	1/27 (4%)	
Any blood product transfusion	36/71 (51%)	20/27 (74%)	0.04

Values are reported as n/N (%) or median (IQR).

APTT, activated partial thromboplastim time; INR, international normalised ratio; PT, prothrombin time.

### Bleeding outcomes

Among the 28% (95% CI 19% to 38%) of neonates with abnormal bleeding, the most common sites of bleeding were pulmonary (23%), upper gastrointestinal (21%) and umbilical (18%) (see online supplementary figure S1). Three neonates had haemorrhage as a contributing cause of death (table 1). Two of these neonates had refractory shock. One of these neonates had a large and expanding subgaleal bleed and the second neonate had a decrease in haematocrit of 19% within 24 hours and required massive transfusion. On autopsy, this second infant was found to have multiple areas of intra-abdominal haemorrhage (spleen, adrenal, liver, ileum). A third patient had significant pulmonary haemorrhage with respiratory deterioration, despite increased positive-end expiratory pressure and endotracheal epinephrine.

10.1136/bmjopen-2016-013787.supp1 supplementary data

**Figure 1:**
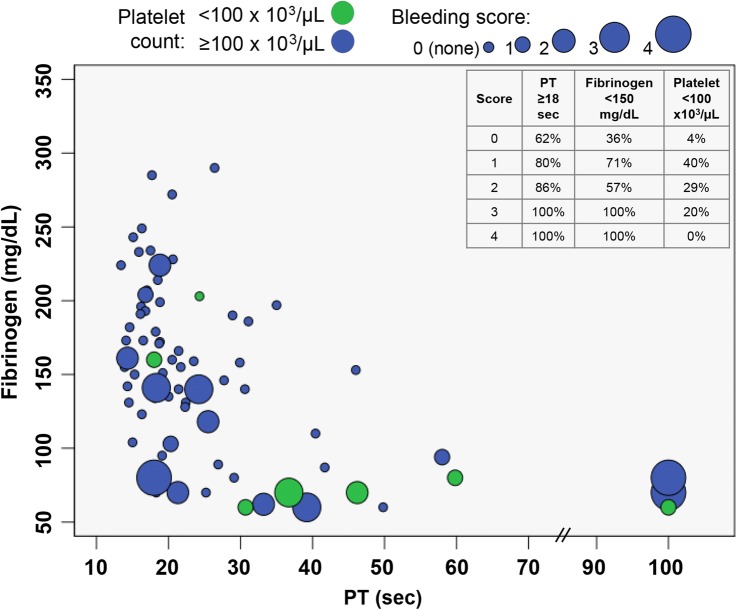
Relationship between haemostatic parameters and bleeding severity. Bubble plot demonstrates initial haemostatic parameters among 97 patients. The size of the bubbles increases with increasing bleeding severity, determined using the WHO bleeding scale and ascertained using the Neonatal Bleeding Assessment Tool (NeoBAT). One infant with an initial fibrinogen of 1400 mg/dL and bleeding score of 2 not shown. The frequency of abnormal haemostatic parameters by bleeding severity score is reported in the inset box.

The per cent of neonates with abnormal bleeding events and the receipt of any blood products increased with increasing PT prolongation (p<0.001 for linear trend for both comparisons) (see online supplementary figure S2). Of the 27 patients with an abnormal bleeding event, 10 (37%) patients had a WHO bleeding severity score of 1, 7 (26%) had a score of 2, 5 (19%) had a score of 3 and 5 (19%) a score of 4. The WHO bleeding severity increased with increasing PT and decreasing fibrinogen levels. Twenty per cent of infants with a bleeding severity score of 3 had an initial platelet count <100×10^3^/μL and none of the infants with bleeding severity score of 4 had an initial platelet count <100×10^3^/μL (figure 1).

### Risk factors for bleeding

In a bivariable analysis, the risk of bleeding increased by 22% for every 10 s increase in initial PT (relative risk 1.22; 95% CI 1.15 to 1.30) (table 3). In multivariable analysis, fibrinogen <150 mg/dL (adjusted relative risk 2.41, 95% CI 1.09 to 5.36) and platelet count <100×10^3^/μL (adjusted relative risk 2.23, 95% CI 1.17 to 4.22), but not degree of PT prolongation, were independently associated with abnormal bleeding. Results were similar when evaluating neonates with a fibrinogen <100 mg/dL instead of <150 mg/dL.

**Table 3:** Bivariable and multivariable risk factors for abnormal bleeding

Factor (bivariable)	n/N	RR	95% CI	p Value
PT (categorised)				
<18 s	29/95	1.00	Reference	
18–30 s	42/95	2.30	0.69 to 7.64	0.17
30 or greater s	24/95	5.24	1.69 to 16.3	0.004
PT (per 10 s increase)	95	1.22	1.15 to 1.30	<0.001
APTT (per 10 s increase)	74	1.08	1.05 to 1.12	<0.001
INR (per 1 point increase)	82	1.17	1.06 to 1.30	0.003
Fibrinogen <100 mg/dL	20/83	4.55	2.29 to 9.03	<0.001
Fibrinogen <150 mg/dL	39/83	3.84	1.56 to 9.43	0.003
Platelet <100×10^3^/μL	10/93	3.06	1.74 to 5.39	<0.001
Initial pH (per 1 point increase)	84	0.62	0.16 to 2.38	0.49
HIE (severe vs moderate)	35/98	1.67	0.89 to 3.14	0.11
Placental abruption	23/98	1.63	0.85 to 3.12	0.14
Uterine rupture	12/98	2.05	1.04 to 4.03	0.04
Factor (multivariable)*		**Adjusted RR**	**95% CI**	**p Value**
PT (categorised)				
<18 s	23/77	1.00	Reference	
18 to <30 s	36/77	1.77	0.43 to 7.29	0.43
30 or greater s	18/77	2.69	0.65 to 11.1	0.17
Fibrinogen <150 mg/dL	36/77	2.41	1.09 to 5.36	0.03
Platelet <100×10^3^/μL	8/77	2.59	1.30 to 5.16	0.007

*For multivariable robust Poisson regression model, only 77 of 98 patients with all initial haemostatic parameters measured were included. Area under the curve of 0.81 (95% CI 0.69 to 0.93), obtained from multivariable logistic regression model.

APTT, activated partial thromboplastim time; HIE, hypoxic–ischaemic encephalopathy; INR, international normalised ratio; PT, prothrombin time; RR, relative risk.

Among initial haemostatic parameters evaluated, an initial PT ≥18 s had the highest sensitivity (88%) and lowest specificity (38%) for discriminating neonates with and without abnormal bleeding, while an initial platelet count <100×10^3^/μL had the highest specificity (96%) and lowest sensitivity (27%) (see online supplementary table S1). We also evaluated the ability of previously published cut-points^5^ to discriminate bleeding in our cohort and found similar performance characteristics for fibrinogen <154 mg/dL, but not platelet count <130.5×10^3^/μL or INR >1.98.

## Discussion

Initial haemostatic dysfunction is highly prevalent among neonates with moderate-to-severe HIE, and is associated with a high transfusion burden and increased risk of bleeding. The prevalence of haemostatic dysfunction in our cohort was 69%, which is higher than the 18–54% reported in prior published studies.^5^ ^7–9^ Some of the prior studies used a more restrictive definition of coagulopathy, which included the need for clinical bleeding, potentially resulting in underestimation of the actual prevalence of haemostatic dysfunction. Our results demonstrate blood product transfusions with packed red blood cells, platelets and fresh-frozen plasma are common in neonates with moderate-to-severe HIE, among bleeding and non-bleeding neonates.

Transfusions are life saving for certain neonates, but there are no standard guidelines for transfusion practices in term neonates with significant variation among medical providers.^12^ ^13^ Early correction of haemostatic dysfunction with massive transfusion protocols has been associated with improved outcomes in paediatric trauma patients.^15^ ^16^ Although the mechanisms of haemostatic dysfunction in neonates with HIE differ from paediatric trauma patients, they may benefit from a standardised transfusion protocol, given the common utilisation of multiple blood product types. Given the strong association between the severity of haemostatic dysfunction and bleeding risk, we believe that prophylactic transfusions with blood products based on laboratory data alone are reasonable. However, the appropriate transfusion thresholds and whether prophylactic transfusion at these thresholds can prevent serious bleeding is uncertain in this population.

We found that thrombocytopenia and hypofibrinogenaemia were independently associated with an increased risk of bleeding. Neonates with lower WHO bleeding scale scores (1 or 2) were more likely to have thrombocytopenia compared with neonates with more severe bleeding, suggesting a disruption of primary haemostasis. In contrast, coagulopathy and hypofibrinogenaemia were more common among neonates with more severe bleeding (scores of 3 or 4), suggesting a problem of secondary haemostasis. Although platelet count <100×10^3^/μL was a predictor of abnormal bleeding, most patients with bleeding had platelet counts >100×10^3^/μL. In the setting of moderate-to-severe HIE, where decreased pH and hypothermia are present, platelet dysfunction despite a normal platelet count may impact the risk of bleeding. The use of cryoprecipitate was relatively infrequent in our study, despite the common finding of hypofibrinogenaemia, particularly among neonates with higher bleeding severity. This contrasts the nearly 40% use of cryoprecipitate in another report.^5^ Cryoprecipitate has a higher fibrinogen content than fresh-frozen plasma. Additional studies are necessary to determine whether early cryoprecipitate use is superior to fresh-frozen plasma to prevent bleeding in this population. In addition, our study highlights the importance of systematically evaluating neonates with HIE for initial haemostatic dysfunction, given 74% of infants received initial haemostatic evaluation, despite adherence to whole body hypothermia protocols that were continued following participation by the two study hospitals in the initial hypothermia trials by the Neonatal Research Network. Of note, approximately half the infants excluded had initial haemostatic evaluation performed after 12 hours of age. Initial haemostatic testing can help identify neonates at higher risk for bleeding complications who could potentially benefit from prophylactic blood product transfusions. Further studies are needed to evaluate transfusion practices, including the relationship between cryoprecipitate transfusion versus fresh-frozen plasma transfusion on bleeding risk and other important outcomes among neonates with moderate-to-severe HIE. Given many of the infants with bleeding received multiple blood products, the role of balanced ratios of transfusion in this population may warrant additional study.

Our study has several strengths. We were able to evaluate a relatively large number of patients at two centres, given our large referral base for neonates with HIE. We were able to quantify bleeding in our patients using the NeoBAT, a standardised bleeding assessment tool for high-risk neonates with high inter-rater reliability when applied in the clinical setting. In addition, this is the first study that has systematically evaluated initial haemostatic dysfunction in patients with moderate-to-severe HIE and the relationship between these parameters and the severity of bleeding.

Our study has several limitations. Since this was a retrospective study, we relied on clinician-ordered haemostatic testing and not all neonates underwent laboratory testing or had testing of all haemostatic parameters. As a result, our results may be biased because haemostatic testing could have been more commonly obtained in neonates with clinical evidence of multisystem organ dysfunction or greater illness severity. However, we found no difference in the severity of HIE, receipt of therapeutic hypothermia or frequency of bleeding between neonates with and without baseline haemostatic testing. In addition, it is possible that haemostatic dysfunction could have been the result of bleeding. To address this, we only included testing that was performed within the first 12 hours of birth and we only evaluated bleeding events for the first 96 hours after birth to reduce the possibility that bleeding was the cause of haemostatic dysfunction or temporally unrelated to initial haematological testing. In addition, haematological parameters in some neonates were obtained following the start of therapeutic hypothermia. However, a prior study suggests this is unlikely to have a significant effect on coagulation parameters.^17^ Furthermore, we could not prospectively characterise bleeding in each infant, which may limit the utility of the NeoBAT in ascertaining bleeding severity.

In conclusion, initial haemostatic dysfunction is prevalent among patients with moderate-to-severe HIE and the severity of haemostatic dysfunction is associated with an increased risk of the frequency and severity of bleeding. These findings indicate that assessment of initial haemostatic dysfunction should be performed routinely in all neonates with moderate-to-severe HIE.
