# Supplementary material for: Observational study of haemostatic dysfunction and bleeding in neonates with hypoxic–ischaemic encephalopathy
Source: BMJ Open. 2017 Feb 9;7(2):e013787. doi: 10.1136/bmjopen-2016-013787 (PMC5306531; doi:10.1136/bmjopen-2016-013787)
Supplement: supplementary data [file bmjopen-2016-013787supp.pdf]

SUPPLEMENTAL DATA

Supplemental Table 1. Discrimination of bleeding vs. non-bleeding neonates by initial hematologic parameters

Supplemental Figure 1. Site of abnormal bleeding events.

Supplemental Figure 2. Abnormal bleeding and transfusion burden among neonates by severity of coagulopathy.

Supplemental Table 1. Discrimination of bleeding vs. non-bleeding neonates by initial hematologic parameters

Study cut-points[a]	State	No bleeding	Bleeding	Sensitivity	Specificity	LR+	LR-	AUC
Fibrinogen < 150mg/dL	Yes	22	17	77%	64%	2.1	0.4	0.706
	No	39	5					
Fibrinogen < 100mg/dL	Yes	7	13	59%	89%	5.1	0.5	0.738
	No	54	9					
Platelet < 150x10^3/μL	Yes	14	12	46%	80%	2.3	0.7	0.629
	No	55	14					
Platelet < 100x10^3/μL	Yes	3	7	27%	96%	6.0	0.8	0.612
	No	64	19					
PT ≥ 18 seconds	Yes	43	23	88%	38%	1.4	0.3	0.631
	No	26	3					
**Published cut-points[b]**	**State**	**No bleeding**	**Bleeding**	**Sensitivity**	**Specificity**	**LR+**	**LR-**	**AUC**
Fibrinogen < 154mg/dL	Yes	25	17	77%	59%	1.9	0.4	0.681
	No	36	5	*(71%)*	*(69%)*			*(0.695)*
Platelet < 130.5x10^3/μL	Yes	11	11	42%	84%	2.7	0.7	0.632
	No	58	15	*(71%)*	*(62%)*			*(0.695)*
INR > 1.98	Yes	24	12	60%	61%	1.6	0.7	0.606
	No	38	8	*(73%)*	*(54%)*			*(0.666)*

Abbreviations: LR, likelihood ratio; AUC, area under the curve; PT, prothrombin time; INR, international normalized ratio.
[a] Study cut-points for platelets and fibrinogen determined using clinically defined thresholds. PT cut-point was determined using an *a priori* defined threshold (for additional details, please see methods).
[b] Published cut-points based on study by Forman et al. *BMC Pediatrics*. 2014, 14:277. Published measures of sensitivity, specificity and AUC are noted *(in parenthesis)* below values externally validated by current study.

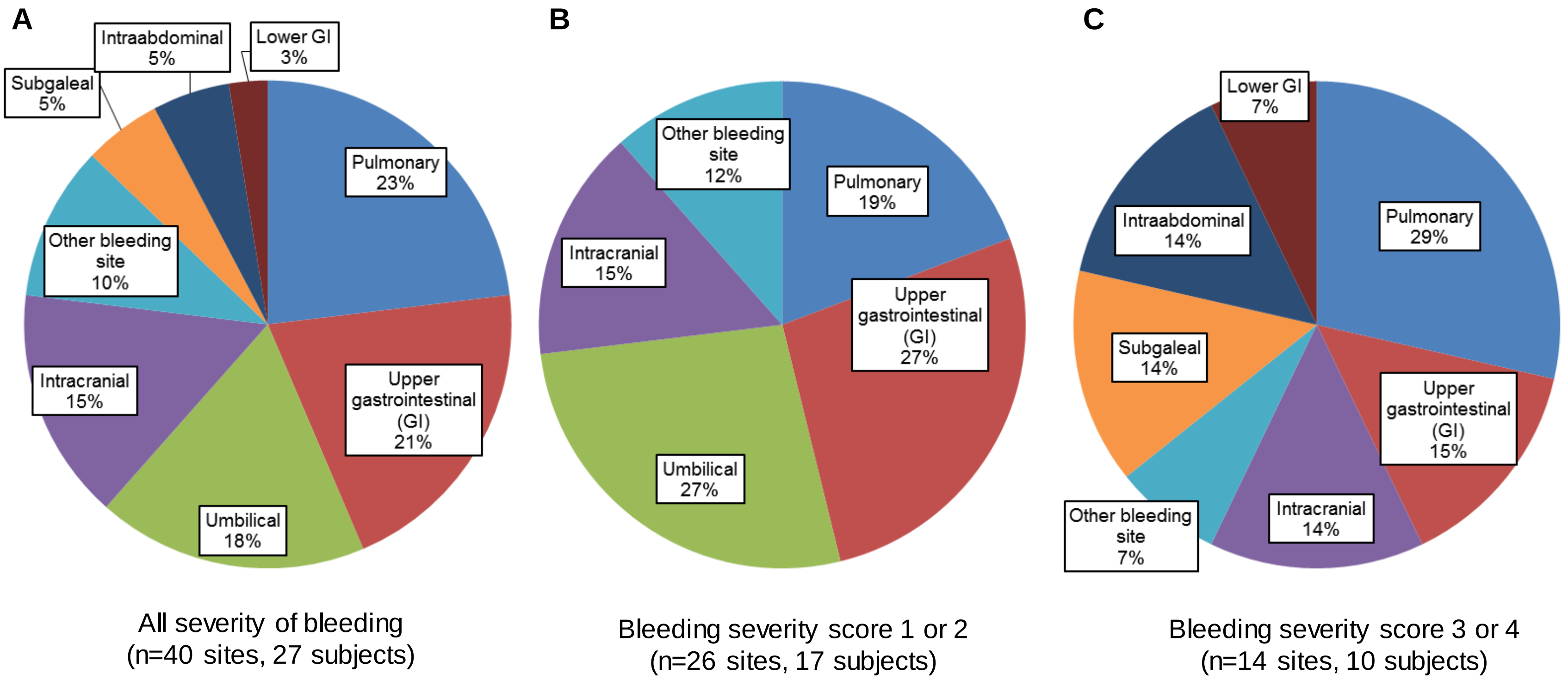

Supplemental Figure 1. Site of abnormal bleeding events. Panel A depicts location of abnormal bleeding sites (n=40) among infants with bleeding (n=27) are shown. Fifteen infants had one bleeding site, 11 infants had 2 bleeding sites, and 1 infant had 3 bleeding sites. Panels B and C depicts bleeding sites by severity of bleeding. No infants with bleeding severity scores 1-2 had subgaleal, intraabdominal or lower GI bleeding. No infants with bleeding severity scores 3 or 4 had umbilical bleeding.

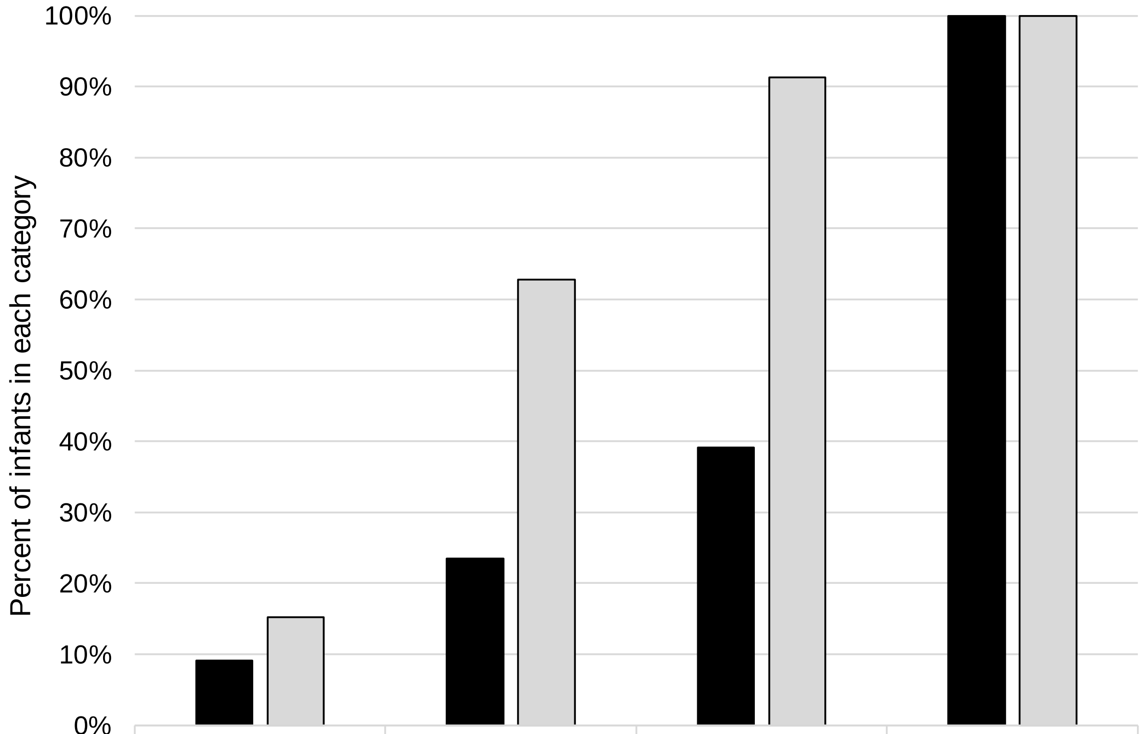

	<18	18 to <30	30 to <90	90 or greater
■ Abnormal bleeding	9%	24%	39%	100%
□ Any blood product transfusion	15%	63%	91%	100%
	n=33	n=51	n=23	n=5

Prothrombin time (sec)

Supplemental Figure 2. Abnormal bleeding and transfusion burden among infants by severity of coagulopathy. Transfusion burden defined as receipt of at least one blood product transfusion of any of the following: packed red cells, fresh frozen plasma, platelets or cryoprecipitate. P<0.001 for linear trend between increasing prothrombin time and both abnormal bleeding and receipt of any blood product transfusion.
